# En-face optical coherence tomography hyperreflective foci of choriocapillaris in central serous chorioretinopathy

**DOI:** 10.1038/s41598-023-33800-0

**Published:** 2023-05-03

**Authors:** Bo-Een Hwang, Joo-Young Kim, Rae-Young Kim, Mirinae Kim, Young-Geun Park, Young-Hoon Park

**Affiliations:** 1 Department of Ophthalmology and Visual Science, Seoul St. Mary’s Hospital, College of Medicine, The Catholic University of Korea, 222 Banpo-daero, Seocho-gu, Seoul, 06591 Republic of Korea; 2 Catholic Institute for Visual Science, College of Medicine, The Catholic University of Korea, Seoul, Republic of Korea

**Keywords:** Retinal diseases, Vision disorders

## Abstract

The purpose of this study is to evaluate choroidal hyperreflective foci (HRF) changes in central serous chorioretinopathy (CSC) on en-face optical coherence tomography (OCT). Retrospective analysis of 42 patients with unilateral CSC (84 eyes, including fellow eyes for controls) and 42 age- and sex-matched controls. With 4.5 × 4.5 mm macular scans, structural en-face OCT choriocapillaris (CC) slabs were used to calculate the density and number of HRF in acute CSC eyes with serous retinal detachment (SRD), resolved CSC eyes without SRD, unaffected fellow eyes, control eyes, and 1-year follow-up eyes. Based on the 2-disc diameter (3000 μm), the en-face OCT scan was divided into foveal and perifoveal lesion and analyzed to consider the impact of SRF in HRF measurement. Regression analyses were performed on the several factors with HRF number and density in the acute and resolved CSC eyes. The perifoveal density and number of CC HRF was significantly lower in the resolved CSC eyes when compared to the acute CSC eyes (*P* = 0.002, both), fellow eyes (*P* = 0.042/density, 0.028/number), and controls (*P* = 0.021/density, *P* = 0.003/number). There was no significant difference between the acute CSC eyes, fellow eyes, controls, and 1-year follow-up eyes. As subfoveal choroidal thickness decreased and choroidal vascularity (CVI) increased, the perifoveal density and number of HRF was measured higher with a significant correlation in univariate regression analysis of the acute and resolved CSC eyes (all, *P* < 0.05). The authors hypothesized that stromal edema induced by choroidal congestion and hyperpermeability has the greatest influence on HRF measurement, possibly affected by inflammatory cells and materials extravasation.

## Introduction

Central serous chorioretinopathy (CSC) is a vision-threatening disease characterized by increased choroidal blood flow and hyperpermeability, causing serous neurosensory retinal detachment (SRD), pigment epithelial detachment (PED), or thickened choroid^1,2^. In the aggravated case of recurrent episodes, prominent retinal pigment epithelium (RPE) alteration could be observed and lead to choroidal neovascularization (CNV) with persistent SRD^3^. Spaide^4^ introduced the starling resistor theory, which can be explained that the structure of noncompliant outershell like brain or eye, that excessively increased blood inflow cannot enlarge vessels anymore, rather constrict the blood flow, leading to maintain suitable volume in the restricted space. In the choroid, vortex vein might perform the role of starling resistor as dilated vortex veins were frequently observed in several CSC studies^4,5^. The constriction of resisting point on vortex vein would contribute to choroidal congestion and increased hydrostatic pressure, causing choroidal hyperpermeability. These choroid physics could raise the question of whether the acute CSC sign; SRD, PED, enlarged choroidal vessel and stroma edema, is a series of severe clinical features, or is it a product of a defense mechanism resulting from the operation of a compensatory mechanism to maintain a constant ocular volume. In addition, there could be another question of whether CSC pathophysiology is explained by the movement of serous fluid from increased hydrostatic pressure and permeability, or whether the inflammation is involved in the disease process and progression.

Hyperreflective foci (HRF) is a characteristic feature that appears in optical coherence tomography (OCT) and en-face OCT, and has already been introduced in several studies^6–11^. In particular, in the study of choroidal HRF in CSC, hypotheses emerged that this would be an exudate of a lipid component or stromal edema^9,12^. In other studies of degenerative retinal diseases and normal eyes, possible candidates of HRF were microglial cells or migrated RPE cells, containing lipids or byproducts of photoreceptors^6,7,11^.

In the present study, to help understand the CSC pathophysiology, we measured continuously the density and number of choriocapillaris (CC) HRF of acute CSC patients using en-face OCT, and obtained clues about HRF identity and choroidal vascular dynamics.

## Methods

### Study population

This retrospective observational study was conducted in the Department of Ophthalmology and Visual Science at Seoul St Mary’s Hospital and adhered to the tenets of the Declaration of Helsinki. All protocols were approved by the Institutional Review Board (IRB) of Seoul St. Mary’s Hospital, The Catholic University of Korea (KC22RISI0493). Owing to the retrospective nature and anonymized data of this study, written informed consent procedures were exempted based on the provisions of the IRB of Seoul St. Mary’s Hospital.

Forty-two patients who were diagnosed with monocular CSC at our clinic were selected to participate in our study; their corresponding fellow eyes and sex-, age-matched control eyes were also studied. All participants were selected between March 2019 and September 2020 at Seoul St. Mary's Hospital in Korea. A retrospective review of their medical records was performed. The exclusion criteria were as follows: (1) refractive errors of more than spherical equivalent of 6 diopters of either myopia or hyperopia; (2) eyes with a history of any ocular trauma, laser treatment, or intraocular surgery; (3) eyes with a history of intravitreal injections for other ocular diseases; (4) other systemic diseases that could affect the retina except hypertension; (5) presence of other retinal diseases, including glaucoma, age-related macular degeneration, diabetic retinopathy, neurodegenerative disease, or other retinal disease affecting macula; (6) media opacity that could affect image quality; and (7) any history of uveitis.

### Study protocol

Demographic data, medical history, and ophthalmologic history were recorded at the initial visit. All subjects underwent an ocular examination, including slit-lamp microscopy, dilated fundus examination, OCT, and OCTA. OCT/OCTA imaging was performed using the Topcon DRI Triton SS-OCT device using a 1050-nm wavelength light source, and a scanning speed of 100,000 A-scans/s. The OCT scan protocol was three-dimensional (3D) scan, which consisted of a number of horizontal line scans covering 12 mm × 9 mm rectangular area. 128 B-scans were scanned in total and each B-scan was consisted of 512 A-scans. The OCTA protocol was also 3D scan, covering macular 4.5 mm × 4.5 mm area (320 pixel × 320 pixel) within the height of 4 mm. Acute CSC was diagnosed with OCT scan images when the patient revealed subretinal fluid (SRF) with neurosensory retinal detachment and choroidal thickening without flat irregular PED. The CSC subjects with SRD, not exceeding 3000 μm diameter in the OCT B-scan, were included in the present study. Fluorescein angiography (FAG), indocyanine green angiography (ICG) were performed to confirm typical acute CSC characteristics of hyperfluorescent leaking and choroidal vessel hyperpermeability without any evidence of CNV. Resolutions of SRD within 6 months were confirmed in all subjects regardless of therapeutic intravitreal injections. The presence of visual symptoms in the past day was included into the exclusion criteria to diagnose the first episode of acute CSC. Scans of unaffected fellow eyes and sex- and age-matched normal controls were also used to be compared with those of the acute and resolved CSC eyes. All en-face OCT images and medical records were reviewed by two experienced independent retinal specialists (Y-H.P. and B-E.H.). Two graders made an agreement about whether CSC patients meet inclusion criteria for the analysis of HRFs.

### Hyperreflective foci and choriocapillaris flow void measurement

**Figure 1:**
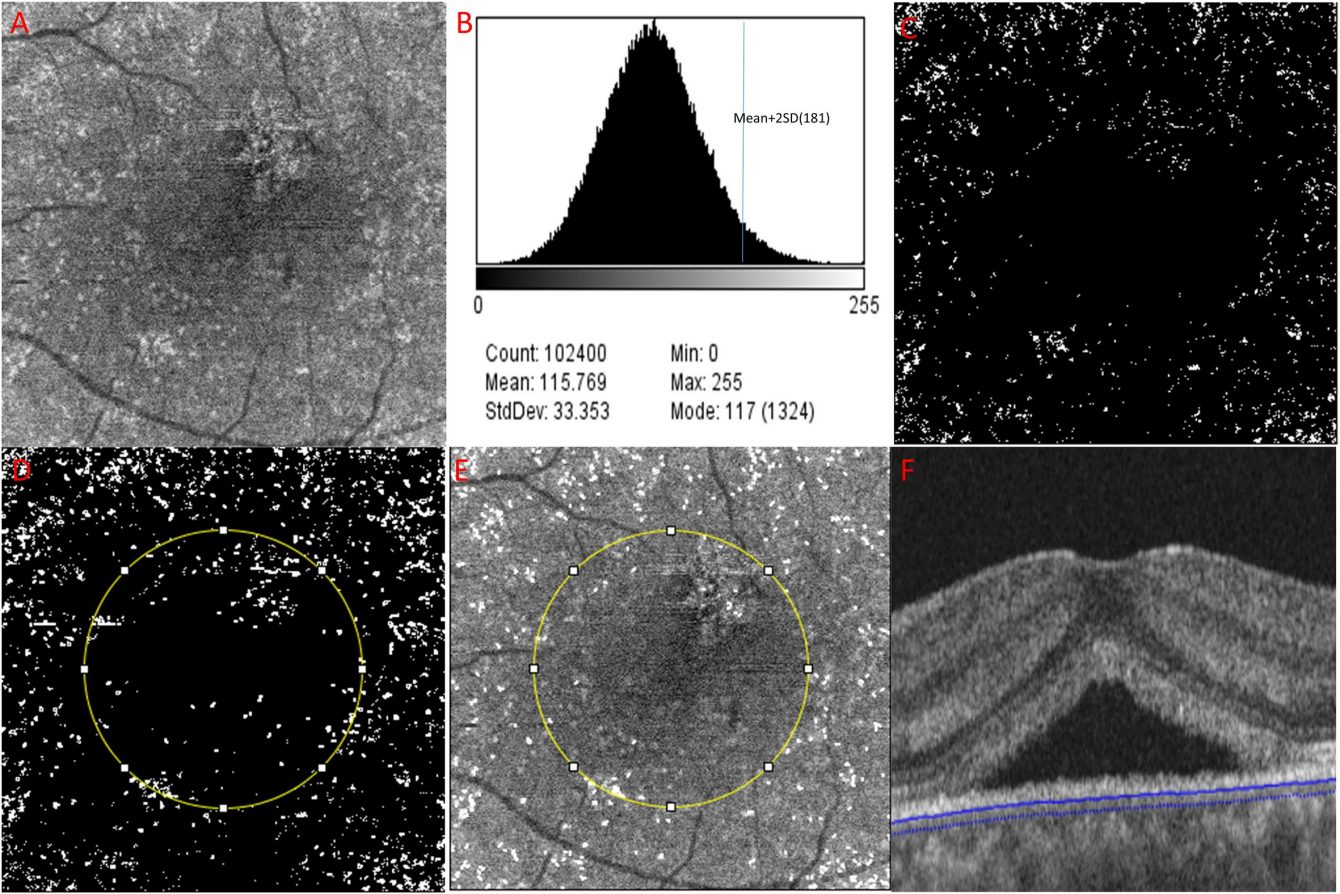
A representative case of acute CSC eyes to demonstrate the process to calculate the number and the density of hyperreflective foci (HRF) in the en-face OCT choriocapillaris (CC) image. (**A**) The 4.5 × 4.5 mm (320 × 320 pixels) en-face OCT images were obtained using a slab from 20.8 micron external to the Bruch’s membrane (BM). (**B,C**) The binarization with a threshold of mean pixel value + 2standard deviation (SD) was applied to the images. (**D**,**E**) Calculated HRF count (n) and the density (%), using Image J tool “Analyze particle” were presented as white in the range of 314–2000 μm. HRFs were measured within the two divided area, foveal and perifoveal lesion, based on 2 discs diameter circular lesion (3000 μm). (**F**) CC images were obtained using a slab from 20.8 micron external to the Bruch’s membrane (BM) in the OCT b-scan.

All en-face OCT images in this study met an image quality of 60 or higher with no line artifacts or noise. Figure 1A–F shows the process of HRF density and number calculation in en-face structural OCT images. The 4.5 × 4.5 mm (320 × 320 pixels) CC images were obtained using a slab from 20.8 micron external to the Bruch’s membrane (BM)^13^, which were calculated from the OCTARA segmentation algorithm built in the Topcon imageNET software (Fig. 1A,F). Considering that the places where fluid, solute, and cellular extravasation due to the increased choroidal permeability would occur the most actively are post capillary venule and capillaris^14^, the values of HRF were measured in the CC layer to understand the physiology of fluid dynamics, inflammatory cell extravasation, or debris from retinal pigment epithelium. The binarization with a threshold of mean pixel value + 2standard deviation (SD) were applied to the images (Fig. 1–C)^6^. Using the “Analyze particle” command in Image J software (version 1.53a; https://imagej.nih.gov/ij/)^15^ that calculated all particles of continuous white pixels in the range of 314–2000 μm, the density (%), count (n) of HRF were measured separately within the foveal and the perifoveal lesion, based on the 2-disc diameter (3000 μm) (Fig. 1D,E)^7^. The reason for analyzing each segment is to consider the effect of detached lesions with SRF on the HRF measurement. We excluded the particle (area) bigger than 2000 μm to minimalize excessively measured errors due to including scar-like RPE fibrotic areas in the HRF calculation. Consider the diameter of the CC and the size of the intercapillary distance^16^, hyperreflective areas larger than 2000 μm were likely to be scar lesion than cells or inflammatory materials. Oh et al.^6^ and Piri et al.^7^ also defined choroidal HRF as round or oval hyperreflective areas with 10–50 μm in diameter. The CC OCTA images were also binarized with the phansalkar threshold method (15 window radius), also using the “Analyze particle” to calculate all threshold areas bigger than or equal to 1 pixel of flow void, the percentage (%) of CC flow void area were also measured^15–17^.

### Choroidal thickness and choroidal vascularity measurement

Choroidal thickness (CT) was calculated using the automatic built-in software within the SS-OCT device. Subfoveal CT was determined by calculating the distance from the outer border of the RPE to the inner edge of the suprachoroidal space^18^. We measured CT manually at the foveal center using digital calipers provided by the SS-OCT software. Using the protocol described by Agrawal et al.^19^, OCT b-scan images were binarized into black and white pixels with a Niblack auto local threshold. Then, the ratio of luminal area (vessel component) to total choroidal area (luminal + stromal) was defined as the choroidal vascularity index (CVI). Two experienced independent observers measured the SFCT and CVI, and the average values were used in the analyses to avoid inter-observer variation.

### Statistical analysis

Statistical analysis was performed using the Statistical Package for the Social Sciences for Windows (version 24.0; SPSS, Inc., Chicago, IL). The mean differences between acute CSC eyes with SRD, resolved acute CSC eye, corresponding fellow eyes, normal control eyes, and 1-year follow-up eyes (n = 20) were assessed using the one-way ANOVA test, followed by post-hoc paired t-test for the HRF density & number. Bonferonni corrections for multiple comparison testing were applied to the significant *P*-value. The intraclass correlation coefficient (ICC) analysis was performed to calculate intergrader reliability. Regression analyses were performed on the several possible relative factors with HRF density and number in the acute CSC eyes and resolved CSC eyes.

### Ethical approval

Owing to the retrospective nature of image analysis and anonymized data of this study, written informed consent procedures were exempted. All protocols were approved by the Institutional Review Board (IRB) of Seoul St. Mary’s Hospital, The Catholic University of Korea (KC22RISI0493).

## Results

**Figure 2:**
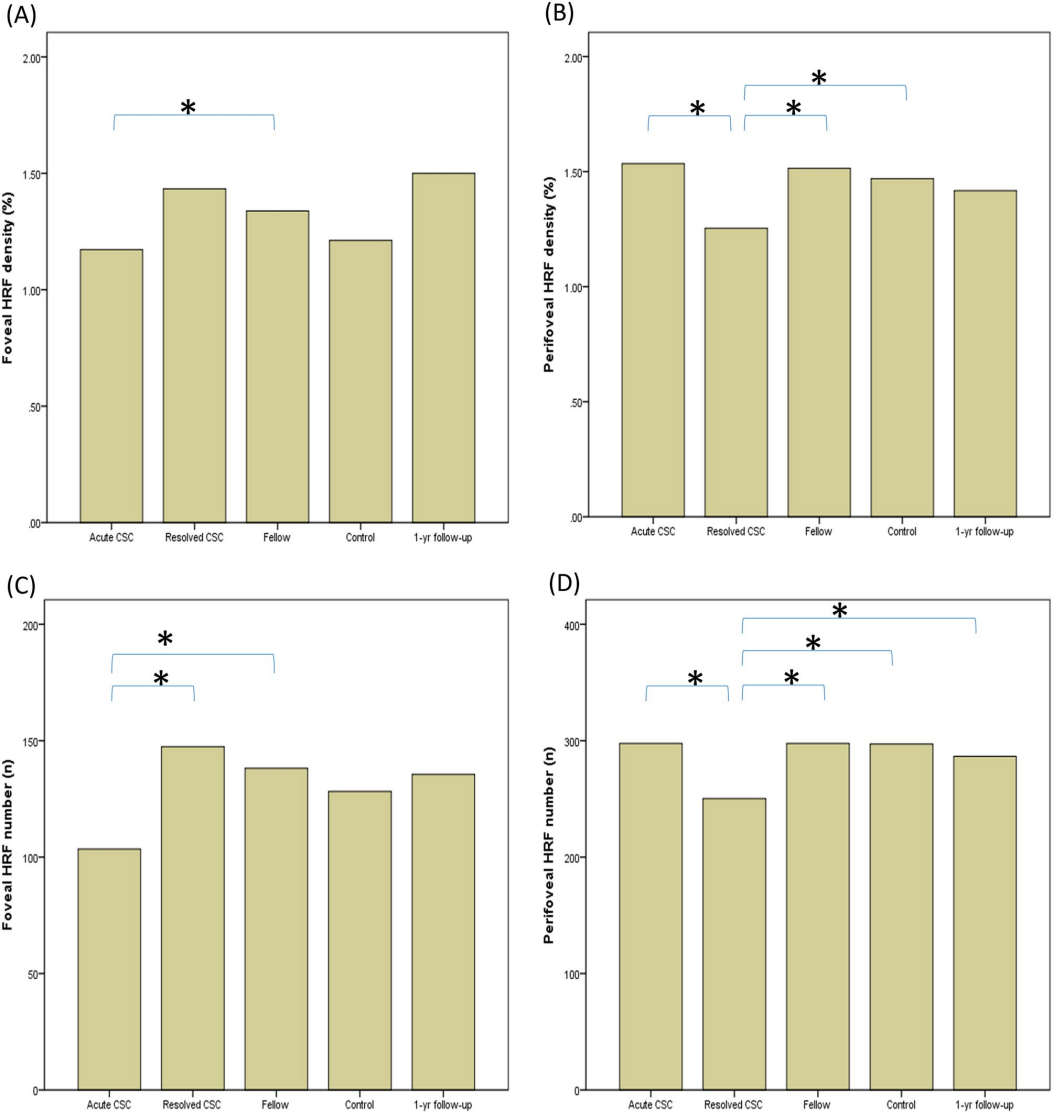
The foveal and perifoveal density (%) and number (n) of HRFs in the en-face OCT choriocapillaris images were presented as bar graph. The mean differences between acute CSC eyes with SRD, resolved acute CSC eye, corresponding fellow eyes, normal control eyes, 1-year follow-up eyes were assessed using the one-way ANOVA test, followed by post-hoc paired t-test for HRF density. The asterisk indicates significant *P*-value after Bonferonni corrections for multiple comparison (< 0.05).

Demographics and characteristics, including initial OCT and OCTA measurements (CT, CVI, CC flow void) are in Table 1. ICC was 0.974 for single measures and 0.987 for average measurements (*P* < 0.0001), which were evaluated as an excellent agreement between two graders^20^. The variation of HRF density (%) and count (n) were statistically significant (*P* = 0.003/density, *P* = 0.001/number) on the comparison of acute CSC eyes with SRD, resolved acute CSC eyes, unaffected fellow eyes, normal control eyes, and 1-year follow-up eyes. On the post hoc analysis, the density and number of perifoveal HRF was lower significantly in the resolved CSC eyes when compared to acute CSC eyes, fellow eyes, controls (*P* = 0.002, 0.042, 0.021/density, *P* = 0.002, 0.028, 0.003/number). The number of perifoveal HRF was significantly higher in 1-year follow-up eyes than in resolved CSC eyes. There was no significant difference between the acute CSC eyes, fellow eyes, controls, and 1-year follow-up eyes (Table 2, Fig. 2).

**Table 1:** Demographics and characteristics of the study subjects.

	CSC (n = 42) (acute/resolved)	Control (n = 42)	*P*-value (acute-resolved/CSC-control)
Age, years	49.48 (± 11.90)	51.90 (± 9.21)	0.636
Sex, Male:female	35:7	35:7	
Disease eye, OD:OS	21:21	21:21	
Hypertension	21.4 (%)	14.3 (%)	0.399
Intraocular pressure	15.29 (± 11.90)	15.00 (± 2.80)	0.286
Refractive error (spherical equivalent)	− 1.36 (± 1.86)	− 1.95 (± 2.39)	0.211
Initial visit VA (LOGMAR)	0.113 (± 0.164)	0.037 (± 0.065)	**0.006**
Initial CT (μm)	461.1 (± 173.0)/427.3 (± 141.9)	265.7(± 86.5)	**0.01/< 0.001**
Initial CVI (%)	63.19 (± 0.95)/62.19 (± 1.39)		** < 0.001**
Initial CC void (%)	39.13 (± 0.72)/38.91 (± 0.75)		0.171

*CC* Choriocapillaris, *CT* Subfoveal choroidal thickness, *CVI* Choroidal vascularity index, *VA* Visual acuity.

Data are presented as the mean (± SD) or a number, as appropriate.

Two-sided *P*-values of < 0.05 were considered to be statistically significant. P-values that are statistically significant are highlighted in bold.

**Table 2:** Hyperreflecitve foci (HRF) Density (%)/Number (n) results.

	Acute (+subretinal detachment) (n = 42)	Resolved (−subretinal detachment) (n = 42)	P value (comparison to acute)	Fellow (n = 42)	*P* value (comparison to acute/resolved)	Control (n = 42)	P value (comparison to acute/resolved)	1-year follow-up (n = 20)	*P* value (comparison to acute/resolved)
**Overall**									
Density	1.38 ± 0.31	1.25 ± 0.29	**0.016 **(0.112)	1.42 ± 0.31	0.427/**0.001 (0.007)**	1.36 ± 0.26	0.799/0.115	1.44 ± 0.33	0.614/**0.006 (0.042)**
Number	403.6 ± 90.4	377.6 ± 81.3	0.112	425.2 ± 77.5	0.165/**0.001 (0.007)**	420.7 ± 61.0	0.327/**0.016 **(0.112)	422.1 ± 94.8	0.441/0.075
**Foveal**									
Density	1.13 ± 0.57	1.27 ± 0.57	0.110	1.39 ± 0.61	**0.006 (0.042)**/0.172	1.18 ± 0.52	0.691/0.499	1.50 ± 0.75	**0.040 **(0.280)/0.441
Number	106.1 ± 52.8	131.4 ± 52.9	**0.006 (0.042)**	142.9 ± 56.2	**0.001 (0.007)**/0.171	124.9 ± 47.4	0.136/0.593	135.5 ± 64.7	**0.027 **(0.189)/0.160
**Perifoveal**									
Density	1.52 ± 0.40	1.24 ± 0.35	** < 0.001 (0.002)**	1.43 ± 0.30	0.231/**0.006 (0.042)**	1.46 ± 0.27	0.462/**0.003 (0.021)**	1.41 ± 0.35	0.206/**0.027 **(0.189)
Number	297.4 ± 77.3	246.3 ± 64.3	** < 0.001 (0.002)**	282.2 ± 57.8	0.311/**0.004 (0.028)**	295.7 ± 46.8	0.902**/< 0.001 (0.003)**	286.6 ± 68.6	0.584/**0.007 (0.049)**

Paired t-test for HRF density & number of acute and resolved central serous chorioretinopathy (CSC) eye, unaffected fellow eye and control eye to analyze the difference between each group. An additional paired T-test was conducted on changes in HRF density & number in 20 patients who were evaluated in the 1-year follow-up visit after subretinal fluid was resolved. Data are presented as the mean (± SD).

Two-sided *P*-values of < 0.05 were considered to be statistically significant. *P*-values that are statistically significant are highlighted in bold.

Bonferonni corrections for multiple comparison testing were applied to the significant *P*-value and corrected *P*-values were added as parenthesis.

**Figure 3:**
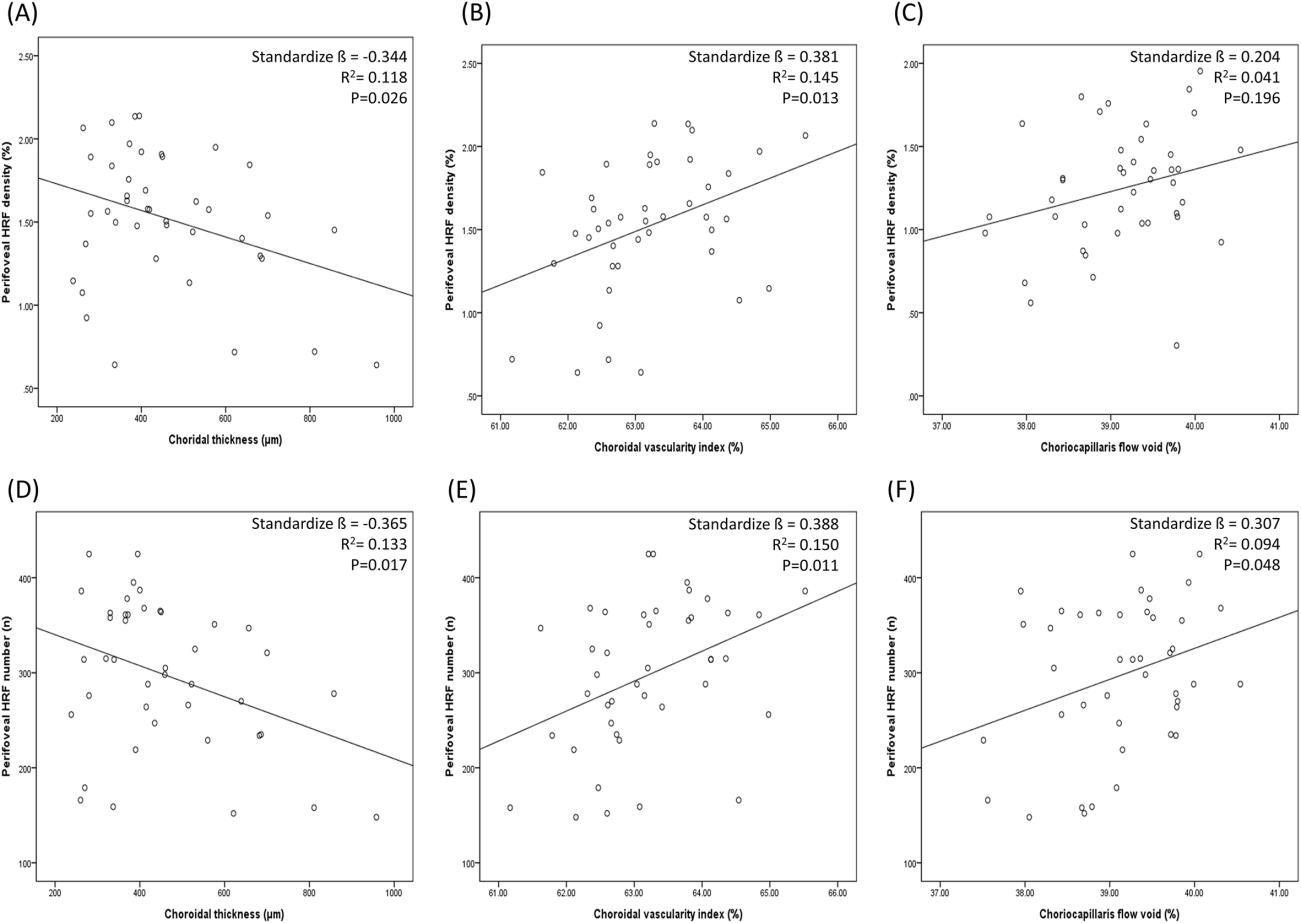
The correlations between perifoveal HRF density, perifoveal HRF number, subfoveal choroidal thickness (SFCT), choroidal vascularity index (CVI), and choriocapillaris (CC) flow void in the acute CSC eyes with subretinal detachment were presented as scatter plots. The value of standardize ß, R^2^, and *P*-value for the regression analysis was shown in the plot.

The results of the linear regression analysis between several possible affecting factors and the HRF density and number in the acute and resolved CSC eyes were presented in Table 3. As SFCT decreased and CVI increased, the density and number of HRF was measured higher with a significant correlation in univariate regression analysis of the acute CSC eyes and the resolved CSC eyes (all, *P* < 0.05). (Table 3, Fig. 3).

**Table 3:** Linear regression analysis of the factors associated with the perifoveal Hyperreflective foci (HRF) density and number.

	HRF density				HRF number			
	Acute		Resolved		Acute		Resolved	
	Standardize ß	*P*-value	Standardize ß	*P*-value	Standardize ß	*P*-value	Standardize ß	*P*-value
Sex (M/F)	0.183	0.246	0.171	0.280	0.219	0.164	0.172	0.275
Age	0.074	0.643	0.349	**0.023**	0.044	0.782	0.296	0.057
OD/OS	0.318	**0.040**	0.257	0.100	0.336	**0.030**	0.262	0.094
Hypertension	0.001	0.997	0.164	0.300	− 0.044	0.782	0.072	0.649
IOP (mmHg)	− 0.106	0.506	− 0.222	0.158	− 0.187	0.236	− 0.246	0.116
Refractive errors (SE)	0.239	0.128	0.055	0.728	0.225	0.151	0.073	0.644
BCVA, logMAR	− 0.006	0.968	0.157	0.319	0.050	0.751	0.119	0.454
Image binarization threshold value	0.164	0.300	− 0.050	0.753	0.126	0.427	− 0.090	0.572
Hx of single Intravitreal injection	0.096	0.546	0.178	0.258	0.102	0.519	0.150	0.342
SFCT (μm)	− 0.344	**0.026**	− 0.365	**0.018**	− 0.365	**0.017**	− 0.394	**0.010**
CVI (%)	0.381	**0.013**	0.338	**0.029**	0.388	**0.011**	0.329	**0.034**
CC flow void (%)	0.204	0.196	0.132	0.404	0.307	**0.048**	0.163	0.301
HRF density in B-scan	− 0.212	0.178	− 0.150	0.344	− 0.172	0.276	− 0.134	0.396
HRF number in B-scan	0.128	0.419	− 0.195	0.096	0.544	0.216	− 0.171	0.278

*ß* Regression coefficient, *BCVA* Best-corrected visual acuity, *logMAR* Logarithm of the minimum angle of resolution, *IOP* Intraocular pressure, *CC* Choriocapillaris, *CVI* Choroidal vascularity index, *SFCT* Subfoveal choroidal thickness, *SE* Spherical equivalent, *Hx* History.

Two-sided *P*-values of < 0.05 were considered to be statistically significant.

*P*-values that are statistically significant are highlighted in bold.

## Discussion

Referring to previous papers, choroidal HRF candidates were summarized as follow; (1) migration of RPE cell with lipid components from retina (photoreceptor)^7,9,11^; (2) stromal localized cell proliferation like microglial cell, melanocyte, fibroblast^6^; (3) exudates or macrophage/monocyte extravasation from increased hydrostatic pressure or inflammation^10,12,21^; (4) chronic fibrotic response^8^. The present study dealt with acute and resolved CSC eyes, the case of (4) might not be a high-probability candidate.

In the study of Oh and colleagues^6^, the number of HRF was higher in the group of lower CVI in the eyes of normal population. It was suggested that the components in the stroma would be HRFs, which were microglial cells, melanocytes, and fibrocytes. In the acute or resolved CSC eyes of the present study, enlarged hydrostatic pressure and choroidal hyperpermeability could contribute to stromal edema. The significant reduction of density and number of HRF at the perifoveal lesion in the resolved CSC eyes might be considered as the dilution of the localized stromal cells, or other inflammatory materials due to sustained stromal edema in the recovery process of CSC. During the SRF resolution, the time difference between the decrease in hydrostatic pressure of congested vessel and the reduction in stromal edema might prevent sudden changes of choroidal volume, which could aggravate the imbalance of autonomic modulation in CSC.

In the acute stage with SRD, we speculated that the increased choroidal blood inflow was combined with choroidal congestion, which would be induced by the starling resistor, maximized choroidal hyperpermeability, accelerating the inflammatory cells or hyperreflective materials extravasation. However, the movement of fluid also considerably aggravated stromal edema, probably not leading to a significantly measured higher in the density and number of HRF in the acute stage, when compared to those of fellow eyes and controls. Sonoda et al. demonstrated that inner choroidal hyperreflective lesion would mean stroma, which was made up of vascular walls, melanocytes, fibrocytes, or neural tissues. They reported that the stromal portion in the inner choroid was significantly larger in CSC eyes than that of fellow eyes and normal control eyes. As the ratio of stroma to the total choroid was relatively lower in CSC^22^, but the absolute amount of stromal edema would be the largest in CSC, especially in the inner choroid, due to the choroidal hyperpermeability, which could contribute to the HRF reduction.

In addition to the dilution effect of the stromal edema on HRF, the authors also focused on lipid or proteinous exudates, and related blood-driven inflammatory cell reactions for the changes of HRF in the present study. In the studies of retinal hyperreflective foci in diabetic retinopathy, HRFs were considered as an inflammation OCT biomarker, especially predicting microglial cell activation and aggregation^23^. As microglia reside in the retina, and tissue-resident macrophage or blood-driven monocyte do the similar roles in the choroid^24^, the alteration of HRF in CC layer from the hemodynamic changes of CSC might be due to the blood-driven inflammatory cell reactions. Regarding lipid exudates, Shinojima et al.^9^ introduced that hyperreflective areas in OCT corresponded ICG-hypofluorescent areas in CSC patients, suggesting that ICG block was due to accumulation of lipid material, or possibly lipid exudates. Sirakaya et al.^25^ presented elevated monocyte to high-density lipoprotein ratio in CSC eyes, which meant increased inflammatory and oxidative stress would be remarkably involved in the CSC pathophysiology. However, if the extravasation of lipid exudates or inflammatory cells do more crucial role than the stromal edema from increased hydrostatic pressure with hyperpermeability in the CSC process, it could not be supported by the result that the higher the choroidal thickness, the less HRF was measured in the regression analysis of our study. Similar results were also found in other HRF studies for the negative correlation between choroidal thickness and HRF^11,12^. In this regard, it was hypothesized that as the large vessel area and the stromal area increases simultaneously, it could be considered that the movement of fluid or solute is much more dominant than the exudation of inflammatory cells or lipid materials.

CVI was known as the parameter for the proportion of choroidal large vessel in Haller’s layer, especially vein^4,19^. The positive correlation between HRF and CVI in the regression analysis was in line with the hypothesis that HRF would come from inflammatory extravasation due to choroidal vein congestion and induced-hyperpermeability, the detrimental effect of which was resolved by photodynamic therapy remarkably as shown in the report of Kinoshita et al^26^. In this respect, the significant correlation result from the perivascular HRF number and CC flow voids of acute CSC (Standardize ß = 0.307, *P* = 0.048) also suggested that HRF increased with hyperpermeability of choroidal vessel despite of the dilution effect of stromal edema. We authors reported the reversible CC flow reduction in acute CSC eye with a novel compensation method^17^. Several studies also demonstrated CC flow delay or hypoperfusion in CSC with choroidal hyperpermeability^5,27,28^. All of these studies supported the significant relevance between choroidal congestion, choroidal hyperpermeability and CC flow reduction. Nevertheless, further research will be needed because the quantitative measurement of CC flow in acute CSC is still challenging and inaccurate.

Piri et el.^7^ observed the HRF in choroids of Stargardt disease, hypothesizing the HRF might be lipofucin materials from outer retina and RPE lesion. In the study of retinal degenerative disease^29^, retinitis pigmentosa, the thicker the choroid, the more HRFs. The opposite results would be because the disease character was different. The HRF of the RP study would be more likely to be lipofucin-related RPE cell migration. In the present study, damaged detached neurosensory retina might induce dysfunction of RPE cell, causing RPE cell migration and lipofucin accumulation in the CC level. Although this hypothesis was not in the line with the result of regression analysis, the result of significant recovery of perifoveal HRF number of 1-year follow-up data would make it possible to speculate that the RPE-lipofucin related mechanism do a partial role in the HRF alteration with the gradual resolution of stromal edema. Through a long-term observational study of the study subjects, the researchers will further clarify the identity of HRFs, focusing on a gradual increasing tendency in chronic process. Additional research, like postmortem or animal studies, will also be needed to clarify this matter.

In the Ruiz-Medrano et al. HRF study, CSC affected eyes, especially chronic ones, tended to have more HRF numbers and hyperreflective fibrous wall thickening^8^. This might be because there was no quantitative restriction on the size when measuring HRF, so the fibrotic changes of RPE from repeated SRD attacks could also appear hyperreflective. It would not be of much help in predicting changes in choroid vessel dynamics when fibrotic changes of RPE were involved in the HRF calculations. If chronic CSC was involved in our HRF study, the results would be uncertain because HRFs would more likely be RPE cell migration with photoreceptor damages from recurrent SRD, resembling degenerative disease, which Arrigo et al.^21^ reported similarly as a high risk factor for macula complications.

Since we planned to focus on the perifoveal lesion that is not affected by SRF during the research planning stage, we delt with the alteration of perifoveal HRFs in the preceding discussions. In fact, even assuming that the effect of SRF on HRF measurements is minimal, the only significant result of lower HRF number in the foveal lesion in the acute CSC would also present the similar the hypothesis we suggested that the excessive stromal edema would occur more largely than hyperreflective inflammatory cell or material extravasation.

The present study would be the prior research, giving opportunity to consider the fluid dynamics and inflammatory process in CSC with the alteration of HRF. However, we acknowledge that the exact histopathological correlate to HRF in CSC was not established and all discussions above rely heavily on hypotheses or conjectures. There were some other limitations to this study. The first was that analysis was only performed in just 4.5 × 4.5 mm sized macular area. Regional variation of vasculature in CSC was found in the report of Kishi et al.^5^, who presented the geographical correspondence of dilated vortex veins and delayed CC flows. If a wider range of area was analyzed with ICG angiography, more valuable evidences would be presented to explain the change in HRF with choroidal fluid dynamics. Second, a comprehensive analysis for the correlation between the en-face choroidal microvasculature HRF and the whole choroidal HRF on B-scan images was not properly conducted in the present study. The reason for analyzing CC slab in this study was that exudates, inflammatory cell extravasation, and hydrostatic fluid shift with the altered permeability would most clearly appear in the CC layer^14^. We additionally calculated the density and number of HRF in the entire choroid by applying the same method on OCT b-scan image. However, any significant correlations between the en-face OCT perifoveal CC HRF and the whole choroidal HRF on b-scan image was not observed (Table 3). In the case of the image of the entire b-scan, the threshold value is set in the entire choroid, in which the inner retina reveals the higher reflectivity than the outer retina as Sonoda et al. reported^10^. For this reason, the reference point will be different from the threshold value set in the macula scan of CC. The difference between the axial and lateral resolution also have contributed the irrelevance when calculated in the same automatic way. These technical limitations require the effective way to standardize the difference between the two different image scans and should be discussed through further research. The third limitation was the suitability of mean + 2SD for the image binarization threshold and the range of 314–2000 μm for HRF size. Another method of comparing RPE reflectivity when defining HRF has also been introduced^29^. It was not apparent to figure out the better automatic method to distinguish whether it is HRF or not in terms of stromal cells, inflammatory cell, or exudates, when only using OCT/OCTA scan and image J tool. Fourth, foveal HRFs were not accurately measured when acute SRD attack occurred. Although we aimed to avoid signal changes from SRF and focused on the perifoveal changes, foveal HRF measurement during acute CSC attack was needed through a more robust method to elucidate CSC pathophysiology more clearly. Fifth, in order to know the exact identity of HRF, these imaging studies will have to be supported by cadaver researches or animal experiments. The unavoidable limitations of image quality in the clinical settings, and the absence of long-term follow up images were other shortcomings in the current study.

In conclusion, although several possibilities remain open for the clear origin of HRF in en-face OCT, we identified the serial changes of HRF in the acute CSC eyes, revealing significantly association with choroidal vascular dynamics. The novel result might give the clinician to get closer to CSC pathophysiology and to consider HRF as parameter for choroidal hyperpermeability and inflammation in eyes with CSC.

## Supplementary Information

Supplementary Information.

## Data Availability

All data generated or analyzed during this study are included in this published article (and its Supplementary Information files).
